# Combined pulmonary fibrosis and emphysema and idiopathic pulmonary fibrosis in non-small cell lung cancer: impact on survival and acute exacerbation

**DOI:** 10.1186/s12890-019-0951-2

**Published:** 2019-10-15

**Authors:** Sung Woo Moon, Moo Suk Park, Young Sam Kim, Joon Jang, Jae Ho Lee, Choon-Taek Lee, Jin-Haeng Chung, Hyo Sup Shim, Kyung Won Lee, Seung-Seob Kim, Sang Hoon Lee, Ho Il Yoon

**Affiliations:** 1 Division of Pulmonology, Department of Internal Medicine, Severance Hospital, Yonsei University College of Medicine, 50-1 Yonsei-ro, Seodaemun-gu, Seoul, 120-752 South Korea; 2 Division of Pulmonary and Critical Care Medicine, Department of Internal Medicine, Seoul National University College of Medicine, Seoul National University Bundang Hospital, Seongnam, Republic of Korea; 3 Department of Radiology, Seoul national University Bundang Hospital, Seongnam, South Korea; 4 Department of Pathology, Yonsei University College of Medicine, Seoul, South Korea; 5 Department of Radiology, Yonsei University College of Medicine, Seoul, South Korea

**Keywords:** Acute exacerbation, Combined pulmonary fibrosis and emphysema (CPFE), Idiopathic pulmonary fibrosis, Mortality, Non-small cell lung cancer

## Abstract

**Background:**

In non-small cell lung cancer (NSCLC) patients, concomitant idiopathic pulmonary fibrosis (IPF) and emphysema (CPFE) are independently related to poor survival. CPFE is a condition with features of both pulmonary fibrosis and emphysema. Here, we evaluated the effect of CPFE and IPF alone on the outcomes of NSCLC patients.

**Patients and methods:**

We retrospectively evaluated 283 patients with CPFE or IPF who were diagnosed with NSCLC between November 2003 and February 2018 at two tertiary care hospitals in South Korea. Patients were classified into CPFE and IPF groups according to chest computed tomography findings.

**Results:**

One-hundred-and-seven patients (37.8%; mean age: 70.1 years; men 97.2%) had CPFE. Compared with IPF patients, CPFE patients had a heavier smoking history; lower diffusing capacity of carbon monoxide (78.0% vs 64.8%, *p* <  0.001), and lower forced expiratory volume in 1 s. Of all patients with NSCLC, 71.7% overall died during the follow-up period; 71.6% died in the CPFE group and 72.0% in the IPF group. Multivariate logistic regression analysis showed that CPFE (odds ratio [OR]: 2.26, 95% confidence interval [CI]: 1.09–4.69; *P* = 0.029) was significantly correlated with acute exacerbations (AEs). In a Cox proportional hazards analysis, stage > III NSCLC, higher Eastern Cooperative Oncology Group performance status, and higher gender–age–physiology index score was related to higher mortality. However, CPFE was not related to a higher mortality rate in univariate (hazard ratio [HR]: 1.00; 95% CI: 0.75–1.32, *P* = 0.972) or multivariate analysis (HR: 0.89; 95% CI: 0.66–1.21, *P* = 0.466).

**Conclusions:**

AE risk, but not all-cause mortality, was higher in patients with CPFE and NSCLC than in those with IPF and NSCLC. Physicians should be aware of the exaggerated risk of AE in patients with concomitant CPFE and NSCLC.

## Background

Idiopathic pulmonary fibrosis (IPF) is a chronic, progressive fibrosing interstitial pneumonia, characterized by progressively worsening dyspnea and lung function, and is associated with a poor prognosis [1]. IPF is reportedly associated with an increased risk of lung cancer [2]. Furthermore, in non-small cell lung cancer (NSCLC) patients, concomitant IPF is related to poor survival [2, 3].

Emphysema is defined as an enlargement of the air spaces distal to the terminal bronchioles due to destruction of the tissues comprising their walls, and can result in an obstructive pattern. Emphysema is also associated with an increased risk of lung cancer [4]. Furthermore, the presence of emphysema in NSCLC patients is also related to a poor prognosis [5].

Emphysema and IPF, which have different radiological, pathological, functional, and prognostic characteristics, have long been regarded as separate entities. However, the coexistence of emphysema and pulmonary fibrosis in individuals is being increasingly recognized [6]. In 2005, Cottin et al. first proposed defining a syndrome termed “combined pulmonary fibrosis and emphysema (CPFE),” which is characterized by a heavy smoking history, exercise hypoxemia, upper lobe emphysema and lower lobe fibrosis, unexpectedly subnormal lung volumes, and severe reduction of carbon monoxide transfer [7]. The pathogenesis of CPFE has not yet been fully elucidated. However, emphysema, IPF, and CPFE have common risk factors, such as smoking [6]. The survival rates of patients with CPFE are known to be poor [8, 9]. Several studies [6, 10–17] have evaluated the clinical course and complications of CPFE. Among them, some reports have stated that patients with CPFE have a higher risk of lung cancer development and death compared with emphysema patients [11, 16]. Nevertheless, the exact clinical course and complications of CPFE are unclear, especially when the condition is comorbid with lung cancer.

As the clinical course and complications of CPFE are not fully understood, physicians may be more reluctant to treat CPFE patients with concomitant NSCLC (CPFE-NSCLC) [17]. Therefore, we aimed to evaluate whether (1) CPFE-NSCLC patients are at higher odds of developing acute exacerbations (AEs) than are IPF patients with NSCLC (IPF-NSCLC) and whether (2) CPFE-NSCLC patients are at higher risk of mortality than are IPF-NSCLC patients.

## Methods

### Study design and population

The study was conducted retrospectively in two tertiary hospitals in South Korea. We evaluated all chest computed tomography (CT) scans of patients with a diagnosis of IPF and lung cancer that were obtained in the Severance Hospital and Seoul National University Bundang Hospital between November 2003 and February 2018. The inclusion criteria were as follows: (1) fulfilment of diagnostic criteria of IPF and CPFE, (2) availability of CT scan images at the time of diagnosis in the institutional radiology database; and (3) availability of clinical data from medical records. In total, 435 patients were considered after applying the inclusion criteria. Among these, patients were excluded if they (1) were diagnosed with small cell lung cancer (*n* = 59); (2) had non-confirmed pathology (*n* = 17); (3) had incomplete data available (*n* = 76); or (4) were lost to follow-up (*n* = 20). Finally, 283 patients were included in the analysis.

Clinical and laboratory data were collected retrospectively from medical records. Data on age, smoking history, pulmonary function test results, underlying diseases, height and weight at the time of the diagnosis, gender–age–physiology (GAP) index score [18], Eastern Cooperative Oncology Group performance status (ECOG) [19], histological type of NSCLC, and clinical and/or pathologic staging of NSCLC were collected for all patients. Information on the treatment modality used for NSCLC; date of treatment; AE after surgery, chemotherapy, or radiotherapy (including the date AE was confirmed); and mortality data (including mortality due to AE) was also collected for all patients. According to smoking status, patients were categorized into two groups (never-smoker vs. ever-smoker [a person who had smoked at least 100 cigarettes and cigars during the course of their life]). The GAP score was calculated based on gender (0–1 points), age (0–2 points), forced vital capacity (FVC) (0–2 points), and diffusing capacity of carbon monoxide (DLco) (0–3 points), and was classified into stages I (0–3 points), II (4–5 points), or III (6–8 points), as previously described [18]. Early NSCLC was defined as stage I or II, while advanced NSCLC was defined as stage III or IV according to the eighth edition lung cancer stage classification [20]. The primary outcome was the development of AE and overall survival. Overall survival was estimated from the date of diagnosis of NSCLC and IPF/CPFE until death. Death registration data were provided by the Ministry of Security and Public Administration of Korea.

### Diagnostic criteria for IPF, CPFE, and AE

IPF was diagnosed using the criteria for the usual interstitial pneumonia (UIP) pattern as described in an official ATS/ERS/JRS/ALAT statement [21], as follows: subpleural, basal, predominantly reticular abnormality or honeycombing, with or without traction bronchiectasis, and the absence of an inconsistent UIP pattern. Diagnosis was confirmed at each hospital by a multi-disciplinary team consisting of specialists in pulmonary medicine, radiology, and pathology.

CPFE was defined according to Cottin et al.’s and Ryerson et al.’s definitions, namely the presence of classic features of centrilobular and/or paraseptal emphysemas (≥ 10%) in the upper lobes and pulmonary fibrosis (mainly IPF/UIP) in the lower lobes radiographically [7, 13]. Classification of CPFE and IPF was based on radiologic findings on chest CT scans. All CT scans of the included patients were reviewed by two radiologist and four pulmonologists, independently.

AE was defined according to the International Working Group Report by Collard et al. [22]: previous or concurrent diagnosis of IPF, acute worsening or development of dyspnea, typically < 1 month in duration, and CT scan with new bilateral ground-glass opacity and/or consolidation, with the deterioration not fully explained by cardiac failure or fluid overload. In the present study, acute exacerbation of IPF or CPFE within 1 month after treatment (chemotherapy, surgery, or radiotherapy) was defined as AE, in order to evaluate the effect of CPFE on the treatment of NSCLC.

### Statistical analysis

Baseline characteristics of CPFE-NSCLC and IPF-NSCLC patients were compared using an unpaired t-test for continuous variables or χ2 test for categorical variables and are presented as mean ± standard deviation and numbers (percentage). Multiple logistic regression models were used to estimate odds ratios (ORs) for AE. Survival times were estimated using the Kaplan–Meier method and compared with the log-rank test. Multivariate Cox proportional hazards models were performed to investigate the relationships between clinical parameters and mortality. Variables that overlapped (e.g., age, gender, FVC, and DL_CO_ in the GAP index) were excluded in the multivariate Cox proportional hazards models. An adjusted *P*-value < 0.05 was considered statistically significant. All statistical analyses were performed with SPSS version 23.0 (SPSS Inc., Chicago, IL, USA).

### Ethics statement

This research protocol was approved by the Institutional Review Board of Severance Hospital, South Korea (IRB No. 4–2018-0770). The study design was approved by the appropriate ethics review boards. The requirement to obtain informed patient consent was waived.

## Results

### Baseline characteristics

Baseline characteristics of the study subjects are provided in Table 1. Men constituted 95.1% of the study cohort. The mean age for the patients overall was 70.3 years (range, 46–89 years); the mean ages of CPFE and IPF patients were 70.4 years (range, 46–89 years) and 70.1 years (range, 51–87 years), respectively. Compared with IPF patients, CPFE patients had a heavier smoking history, lower DLco (78.0% vs. 64.8%, *P* <  0.001), and lower forced expiratory volume in 1 s (FEV_1_)/FVC ratio (75.1% vs. 71.2%, *P* = 0.001). Body mass index (BMI), FVC, NSCLC histology, FVC predicted %, FEV_1_ predicted %, NSCLC stage, ECOG status, treatment modality, and GAP score did not differ significantly between IPF and CPFE patients.

**Table 1:** Patient characteristics according to the presence of CPFE

Variable	IPF-NSCLC	CPFE-NSCLC	*P*-value
Number of patients, n (%)	176 (62.2%)	107 (37.8%)	
Age, years	70.4 ± 8.4 (46–89)	70.1 ± 7.1 (51–87)	0.810
Sex, men	165 (93.8%)	104 (97.2%)	0.263
BMI, kg/m^2^	23.4 ± 3.2 (15.2–30.7)	22.9 ± 3.2 (13.3–29.8)	0.222
Ever smoker, %	161 (91.5%)	103 (96.3%)	0.145
Smoking history, pack-years	35.6 ± 23.8 (0–120)	46.4 ± 26.4 (0–180)	< 0.001
Histology, numbers (%)			0.344
Adenocarcinoma	67 (38.1%)	50 (46.7%)	
Squamous cell carcinoma	84 (47.7%)	45 (42.1%)	
Others	25 (14.2%)	12 (11.2%)	
FVC, % predicted	83.3 ± 18.8 (34–125)	82.8 ± 17.4 (41–121)	0.844
FEV_1_, % predicted	90.2 ± 20.7 (5–140)	86.0 ± 18.7 (44–135)	0.091
DL_CO_, % predicted (*n* = 249)	78.0 ± 21.5 (33–128)	64.8 ± 17.9 (16–102)	< 0.001
FEV1/FVC, Percentage	75.1 ± 8.4 (48.2–96.3)	71.2 ± 9.6 (16–102)	0.001
NSCLC Stage, numbers (%)			0.617
Stage I	45 (25.6%)	27 (25.2%)	
Stage II	27 (15.3%)	13 (12.1%)	
Stage III	38 (21.6%)	30 (28.0%)	
Stage IV	66 (37.5%)	37 (34.6%)	
ECOG ≥2, n (%)	38 (21.6%)	16 (15.0%)	0.106
Follow-up time, months	24.1 ± 24.1 (0.0–138.0)	18.6 ± 25.5 (0.3–137.8)	0.975
Time Gap between Diagnosis of IPF or CPFE and Diagnosis of NSCLC, months	16.4 ± 30.7 (0–149.6)	16.1 ± 31.1 (0–148.4)	0.937
GAP Index score	3.17 ± 1.19 (1–8)	3.38 ± 1.21 (1–7)	0.166
Received chemotherapy, n (%)	78 (44.3%)	53 (49.5%)	0.461
Received operation, n (%)	73 (41.7%)	37 (34.9%)	0.313
Received radiotherapy, n (%)	24 (13.6%)	20 (18.7%)	0.310
AE, n (%)^a^	19 (12.5%)	19 (21.3%)	0.098

Continuous variables are presented as mean ± SD (range) and categorical variables are presented as numbers (percentage)

Definition of abbreviations: BMI, Body Mass Index; CPFE, Combined Pulmonary Fibrosis and Emphysema; NSCLC, Non-Small Cell Lung Cancer; ECOG, Eastern Cooperative Oncology Group performance status; AE, Acute Exacerbation; GAP Score, Gender–Age–Physiology Score; FVC, Forced Vital Capacity; FEV_1_, forced expiratory volume in 1 s; DL_CO_, diffusing capacity of carbon monoxide

^a^AE data could be collected from 241/283 patients

Of the NSCLC patients overall, 71.7% died during the follow-up period; 71.6% died in the CPFE group and 72.0% in the IPF group. The respective follow-up periods were 18.6 months and 24.1 months (*P* = 0.975). CPFE patients had an increased tendency to develop AE, although this was not statistically significant in the univariate analysis (12.5% vs. 21.3%, *P* = 0.098). The time elapsed between diagnosis of CPFE or IPF and NSCLC was analyzed but did not differ significantly between the two groups (16.1 months vs. 16.4 months, *P* = 0.937).

### AE and CPFE

Additional file 1: Table S1 shows the incidences of AE after treatment according to the treatment modality. Nine patients had undergone surgery and adjuvant chemotherapy, 22 patients had undergone concurrent chemoradiation therapy, and four patients had undergone surgery, followed by concurrent chemoradiation therapy. All AE were calculated separately after the respective treatments. NSCLC patients with CPFE tended to have more AE after surgery (IPF 8.6% vs. CPFE 21.6%, *P* = 0.073). Although the incidence of AE was higher in the CPFE group (13.1% vs. 21.3%, P = 0.098), no statistically significant difference was found.

Table 2 shows the relationship between CPFE and AE in the logistic regression model. GAP stage, smoking status, NSCLC histology, NSCLC stage, and CPFE were included in the regression model. In the analysis, due to the small number of patients in the GAP stage III group (*n* = 14), this group was combined with the GAP stage II group. CPFE (OR: 2.26, 95% CI: 1.09–4.69, *P* = 0.029) and GAP stage > II (OR: 2.20; 95% CI: 1.05–4.64; *P* = 0.037) showed a significant correlation with AE. NSCLC histology, smoking history (ever-smoker), and NSCLC stage > III did not show significant correlations with AE.

**Table 2:** Logistic regression analyses of factors related to AE

Variable	OR	95% CI	*p*-value
GAP stage, II and III	2.20	1.05–4.64	0.037
Ever smoker	3.05	0.38–24.23	0.292
Histology			
Adenocarcinoma	1.00		
Squamous cell carcinoma	0.60	0.26–1.36	0.220
Others	1.04	0.35–3.11	0.950
NSCLC stage ≥ III	1.85	0.83–4.16	0.135
CPFE	2.26	1.09–4.69	0.029

Values are presented as odds ratios (ORs) with 95% confidence intervals (CIs)

*Definition of abbreviation*: *GAP Score* Gender–Age–Physiology Score, *NSCLC* Non-Small Cell Lung Cancer, *CPFE* Combined Pulmonary Fibrosis and Emphysema

### Survival and CPFE

**Fig. 1:**
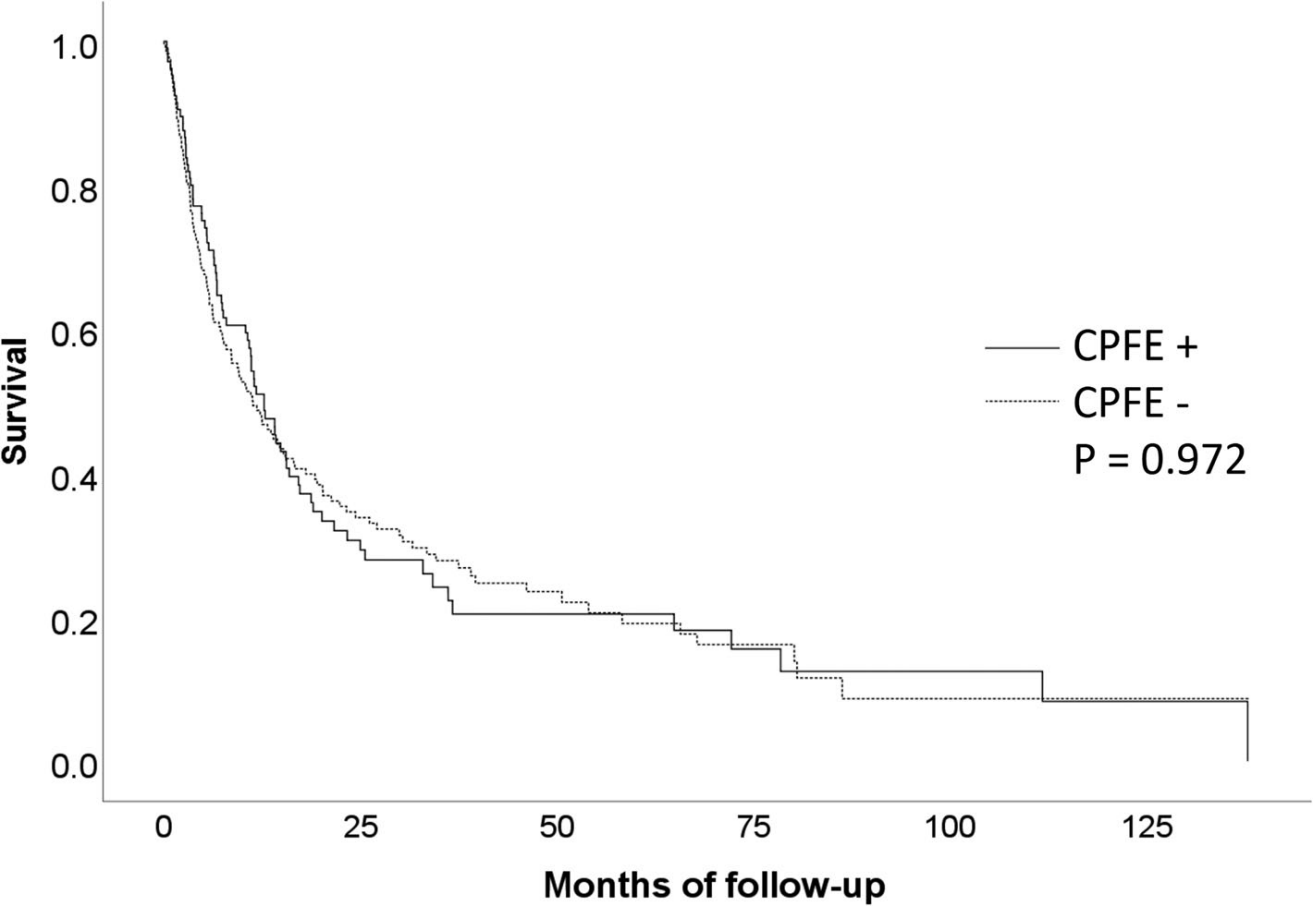
Kaplan–Meier survival curves stratified by the presence of combined pulmonary fibrosis and emphysema (CPFE)

There was no significant difference in survival rates between the IPF and CPFE groups (*P* = 0.972), according to analysis of the Kaplan–Meier survival curves (Fig. 1).

The relationships between all-cause mortality and clinical parameters, including CPFE, were evaluated using Cox proportional hazards analysis (Table 3). Univariate analysis showed that advanced stage NSCLC, higher GAP index score (*P* <  0.001), AE (P <  0.001), lower FVC predicted, lower FEV1 predicted, lower DLco predicted, higher FEV1/FVC, %, and histological type other than adenocarcinoma or squamous cell carcinoma were significantly correlated with all-cause mortality. BMI, smoking history, time elapsed between diagnosis of IPF or CPFE and diagnosis of NSCLC, and CPFE did not correlate with all-cause mortality.

**Table 3:** Clinical factors associated with all-cause mortality (univariate analysis)

Variables	HR	95% CI	*P* value
BMI, kg/m^2^	0.92	0.88–0.96	< 0.001
Ever Smoker, Yes	0.897	0.55–1.46	0.897
CPFE, yes	0.995	0.75–1.32	0.972
NSCLC Stage ≥ III	3.68	2.69–5.03	< 0.001
ECOG	1.44	1.24–1.68	< 0.001
AE, Yes	2.91	1.73–4.91	< 0.001
GAP Score	1.50	1.33–1.70	< 0.001
Sex, Male	0.971	0.54–1.74	0.920
FVC, % Predicted	0.98	0.98–0.99	< 0.001
FEV_1_, % Predicted	0.97	0.96–0.98	< 0.001
DLco, % Predicted	0.98	0.98–0.99	< 0.001
FEV_1_/FVC, %	1.03	1.01–1.04	0.004
Time Gap between Diagnosis of IPF or CPFE and Diagnosis of NSCLC, months	1.00	1.00–1.01	0.265
Histologic type			
Adenocarcinoma	1.00		
Squamous cell carcinoma	0.93	0.69–1.26	0.647
Others	1.68	1.10–2.55	0.016

Data are presented as hazard ratios (95% confidence intervals)

*Definition of abbreviations*: *BMI* Body Mass Index, *CPFE* Combined Pulmonary Fibrosis and Emphysema, *NSCLC* Non-Small Cell Lung Cancer, *ECOG* Eastern Cooperative Oncology Group performance status, *AE* Acute Exacerbation, *GAP Score* Gender–Age–Physiology Score, *FVC* Forced Vital Capacity, *FEV*_*1*_ forced expiratory volume in 1 s, *DL*_*CO*_ diffusing capacity of carbon monoxide

Multivariate Cox proportional hazards analyses were performed to compare the contributions of these indices (Table 4). Stepwise Cox proportional hazards analysis demonstrated that higher ECOG status (HR: 1.30; 95% CI: 1.16–1.55; *P* = 0.003), advanced stage NSCLC (HR: 3.15; 95% CI: 2.21–4.49; P <  0.001), and higher GAP score (HR: 1.31; 95% CI: 1.16–1.48; P <  0.001) were risk factors for all-cause mortality. CPFE (HR: 0.89; 95% CI: 0.66–1.21; *P* = 0.466) was not a significant risk factor for all-cause mortality in multivariate analysis.

**Table 4:** Clinical factors associated with all-cause mortality (multivariate analysis)

Variables	HR	95% CI	*P* value
BMI, kg/m^2^	0.99	0.95–1.04	0.775
CPFE, yes	0.89	0.66–1.21	0.466
NSCLC Stage ≥ III	3.15	2.21–4.49	< 0.001
ECOG	1.30	1.09–1.55	0.003
GAP Score	1.31	1.16–1.48	< 0.001
Histologic type			
Adenocarcinoma	1.00		
Squamous cell carcinoma	0.97	0.70–1.34	0.849
Others	1.27	0.80–1.99	0.309

Data are presented as hazard ratios (95% confidence intervals)

*Definition of abbreviations*: *BMI* Body Mass Index, *CPFE* Combined Pulmonary Fibrosis and Emphysema, *NSCLC* Non-Small Cell Lung Cancer, *ECOG* Eastern Cooperative Oncology Group performance status, *GAP Score* Gender–Age–Physiology Score

## Discussion

In this study, we have described differences in clinical features and outcomes between NSCLC-IPF and NSCLC-CPFE. Although previous studies have compared CPFE to IPF, few studies have compared CPFE-NSCLC to IPF-NSCLC. We demonstrated that CPFE is related to AE, but is not a significantly greater risk factor of all-cause mortality compared with IPF in NSCLC patients.

In this study, the prevalence of CPFE was 37.8% in patients with NSCLC and pulmonary fibrosis. In previously reported studies, the proportion of patients with CPFE detectable on a high-resolution CT scan ranged from 8 to 51% in IPF patients [13, 23]. As the prevalence of lung cancer in CPFE is reported to be higher than that in IPF patients (50% vs. 14.5%) [24], the prevalence reported in this study is in accordance with the findings of previous studies. Cigarette smoking is the main risk factor for NSCLC, IPF, and emphysema, and IPF and emphysema are additional independent risk factors for NSCLC; thus, it is plausible that CPFE patients have a heavier smoking history and higher prevalence of NSCLC. The average smoking pack-years were higher in the CPFE group than in the IPF group in our study. These conditions also share many pathogenic pathways, including genetic and epigenetic alterations, tissue invasion, uncontrolled proliferation, and activation of specific signal transduction pathways [25].

We assessed the median duration from the diagnosis of CPFE/IPF and diagnosis of NSCLC in patients who developed CPFE/IPF prior to developing NSCLC. The duration did not differ between the CPFE and IPF groups (20.01 months vs. 21.06 months, *P* = 0.618). This may suggest that CPFE patients do not necessarily need a shorter follow-up period compared with IPF patients to monitor for the presence of lung cancer. The result did not differ when the patients who were diagnosed concurrently (i.e., diagnosed with NSCLC within 1 month before or after the diagnosis of IPF or CPFE) were excluded (43.0 months vs. 38.5 months, *P* = 0.670).

The annual incidence of AE in patients with IPF has been reported as 5–15% [26]. Additionally, the incidence rate of AE triggered by chemotherapy [27], surgery [28], and radiotherapy [29] is increased in patients with IPF. In our study, the total incidence of AE was 15.7%. We found that CPFE was an independent risk factor of AE after treatment of NSCLC, similar to advanced IPF (higher GAP stage). In a retrospective study of 487 patients who underwent lobectomy for lung cancer, Saito et al. found that seven of 10 post-lobectomy acute respiratory distress syndrome cases (70%) had CPFE [30]. In Japan, Minegishi et al. have reported that the incidence of AE associated with anticancer treatment in lung cancer patients with interstitial lung disease is 10–30% for surgical resection and 9–21% for chemotherapy [12]. In our study, surgical resection patients with CPFE were at higher odds of developing AEs than were patients with IPF. Our study adds evidence that, regardless of whether invasive or non-invasive treatment is used, CPFE may increase the risk of AE.

In IPF and lung cancer patients, efforts are being made to prevent the development of AE. Iwata et al. showed that perioperative pirfenidone for lung cancer surgery in patients with IPF significantly decreased the incidence of AE after surgery [31]. This may also apply to CPFE patients, but further studies are required.

CPFE did not affect the mortality rate of NSCLC patients, although AE has been associated with increased mortality. AEs of IPF are a well-described complication after lung resection, with an incidence of approximately 15% and mortality of 33.3–100% in some studies [32]. It is possible that, in IPF-NSCLC and CPFE-NSCLC patients, the main prognostic factor is the NSCLC stage. Similarly, Goto et al. [33] showed that IPF is a prognostic factor in stage I/II NSCLC, but not in stage III/IV NSCLC. Another possibility is that the outcomes of AEs of CPFE could be more favorable than those of IPF. Toyoshima et al. [34] have reported that, in a comparison of AEs of CPFE and IPF, the AEs of CPFE had a more favorable outcome. Lastly, it is possible that the general prognosis of CPFE-NSCLC patients is better than that of IPF-NSCLC patients, without AEs. Some studies have indicated a much worse prognosis for CPFE than for IPF [35, 36]. However, there are data indicating that CPFE patients have the same [10] or even longer survival [37] compared with IPF subjects. Further studies are needed on this matter.

Our study had some limitations. First, it included only Korean patients and only two centers participated in the study. Some studies of drug-induced lung injury have suggested ethnic differences in the susceptibility to acute progressive respiratory failure during the course of IPF [38]. Large multiregional prospective studies are needed to eliminate an ethnic bias. Second, there might have been bias in patient selection. Of the patients who met the inclusion criteria, the proportion of patients who had incomplete data or who were lost to follow-up was high (22%). Furthermore, as our study population comprised patients who underwent chest CT scans at a tertiary hospital, the population may not be fully representative of the disease population.

## Conclusions

In conclusion, the risk of AE was higher in patients with CPFE and NSCLC, but all-cause mortality was not higher in NSCLC patients with concomitant CPFE than in those with concomitant IPF. Physicians should be aware of the increased risk of AE when treating NSCLC patients with CPFE. A multidisciplinary approach is required for treating these patients.

## Supplementary information

**Additional file 1:** **Table S1.** Incidence of acute exacerbation according to treatment modality.

## Data Availability

The datasets used and/or analyzed are available from corresponding author upon reasonable request.

## Abbreviations

AE: Acute exacerbation
CPFE: Combined pulmonary fibrosis and emphysema
CT: Computed tomography
DL_CO_: Diffusing capacity of carbon monoxide
ECOG: Eastern Cooperative Oncology Group performance status
FEV1: Forced expiratory volume in 1 s
FVC: Forced vital capacity
GAP: Gender–Age–Physiology
IPF: Idiopathic pulmonary fibrosis
NSCLC: Non-small cell lung cancer
UIP: Usual interstitial pneumonia
