# Supplementary material for: Combined pulmonary fibrosis and emphysema and idiopathic pulmonary fibrosis in non-small cell lung cancer: impact on survival and acute exacerbation
Source: BMC Pulm Med. 2019 Oct 15;19:177. doi: 10.1186/s12890-019-0951-2 (PMC6792261; doi:10.1186/s12890-019-0951-2)
Supplement: Supplementary file 1 — Additional file 1: Table S1. Incidence of acute exacerbation according to treatment modality. [file 12890_2019_951_MOESM1_ESM.doc]

**Additional file**

**TITLE: Combined pulmonary fibrosis and emphysema and idiopathic pulmonary fibrosis in non-small cell lung cancer: Impact on survival and acute exacerbation**

Sung Woo Moon (Fure1984@gmail.com)^a^, Moo Suk Park (PMS70@yuhs.ac)^a^, Young Sam Kim (YSAMKIM@yuhs.ac)^a^, Joon Jang (Chang@yuhs.ac)^a^, Jae Ho Lee (jhlee7@snubh.org)^b^, Choon-Taek Lee (ctlee@snu.ac.kr)^b^, Jin-Haeng Chung (jhchung@snubh.org)^c^, Hyo Sup Shim (Shimhs@yuhs.ac)^d^, Kyung Won Lee (Lkwrad@gmail.com)^c^, Seung-Seob Kim(K2s0127@yuhs.ac)^e^, Sang Hoon Lee (Cloud9@yuhs.ac)^a^, Ho Il Yoon (Dextro70@gmail.com)^b^

**Affiliations:**

^a^Division of Pulmonary and Critical Care Medicine, Department of Internal Medicine, Severance Hospital, Yonsei University College of Medicine, Seoul, Korea

^b^Division of Pulmonary and Critical Care Medicine, Department of Internal Medicine, Seoul National University College of Medicine, Seoul National University Bundang Hospital, Seongnam, Korea

^c^Department of Radiology, Seoul national University Bundang Hospital, Seongnam, Korea

^d^Department of Pathology, Yonsei University College of Medicine, Seoul, Korea

^e^Department of Radiology, Yonsei University College of Medicine, Seoul, Korea

Table S1. Incidence of acute exacerbation according to treatment modality

| Variable | Idiopathic pulmonary fibrosis | Combined pulmonary fibrosis and emphysema | p-value |
| --- | --- | --- | --- |
| Chemotherapy | 11 / 77 (14.3%) | 11 / 53 (20.8%) | 0.351 |
| Surgery | 6 / 70 (8.6%) | 8 / 37 (21.6%) | 0.073 |
| Radiotherapy | 4 / 24 (16.7%) | 5 / 20 (25.0%) | 0.710 |
